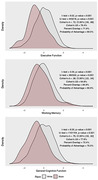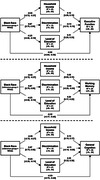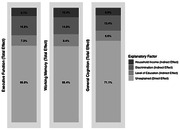# Racial Disparities in Cognitive Function & Mild Cognitive Impairment: Effects of Income, Education, and Discrimination in the Midlife in the United States (MIDUS) Study

**DOI:** 10.1002/alz.086744

**Published:** 2025-01-09

**Authors:** Frank D Mann, Adolfo G Cuevas, Anthony D Ong, Samantha S Corley, Colin D Freilich, Kristian E Markon, Sean A.P. Clouston, Robert F. Krueger

**Affiliations:** ^1^ Stony Brook University, Stony Brook, NY USA; ^2^ New York University, New York, NY USA; ^3^ Cornell University, Ithica, NY USA; ^4^ University of Minnesota, Minneapolis, MN USA

## Abstract

**Background:**

Racial differences in the prevalence of mild cognitive impairment (MCI) and Alzheimer’s Disease and related dementia (ADRD) are well documented in aging populations. Using data from a large longitudinal study of adults, Black‐White disparities in mild cognitive impairment (MCI) were estimated and putative mediators of Black‐White disparities in cognitive function were examined.

**Method:**

The prevalence of MCI was determined algorithmically for Black and White adults, and longitudinal indicators of income, educational attainment, and interpersonal discrimination were used to conduct a parallel mediation analysis of Black‐White disparities in executive function and episodic memory.

**Result:**

As expected, there was a statistically significant racial disparity in the prevalence of MCI, such that MCI was more prevalent among Black adults (6.4%) than White adults (3.0%), which was statistically significant according to Fisher’s exact test (*OR* = 2.20, *p* = .041). Continuous distributions of executive function, episodic memory, and general cognition also differed significantly for Black and White adults (range of Cohen’s d = [0.38, 0.76], p‐values < .01), as did putative mediators of Black‐White disparities in cognitive outcomes, including income, educational attainment, and exposure to discrimination (range of Cohen’s d = [0.26, 1.07], p‐values < .01). Parallel mediation analyses indicated that household income, educational attainment, and interpersonal discrimination were all statistically significant (p‐values < .005) mediators of Black‐White disparities.

**Conclusion:**

Approximately 1/3 of the observed Black‐White disparity in executive function, episodic memory, and general cognition was accounted for by racial group differences in income, education, and cumulative exposure to interpersonal discrimination, which included lack of support from coworkers and supervisors. Conversely, the remaining 2/3 of the Black‐White racial disparity was neither explained by group differences in income, education, nor exposure to discrimination. Future research should focus on identifying additional socioeconomic and modifiable interpersonal factors that account for Black‐White disparities in cognitive dysfunction and mild cognitive impairment in aging populations.